# Long-term total hip arthroplasty rates in patients with acetabular and pelvic fractures after surgery: A population-based cohort study

**DOI:** 10.1371/journal.pone.0231092

**Published:** 2020-04-03

**Authors:** Tzu-Chun Chung, Tzu-Shan Chen, Yao-Chun Hsu, Feng-Chen Kao, Yuan-Kun Tu, Pao-Hsin Liu

**Affiliations:** 1 Department of Orthopaedics, E-Da Hospital, Kaohsiung, Taiwan; 2 School of Medicine for International Students, College of Medicine, I-Shou University, Kaohsiung, Taiwan; 3 Department of Medical Research, E-Da Hospital, Kaohsiung, Taiwan; 4 Department of Medical Imaging and Radiological Sciences, College of Medicine, I-Shou University, Kaohsiung, Taiwan; 5 School of Medicine, College of Medicine, I-Shou University, Kaohsiung, Taiwan; 6 Department of Internal Medicine, E-Da Hospital, Kaohsiung, Taiwan; 7 Graduate Institute of Clinical Medical Science, China Medical University, Taichung, Taiwan; 8 Department of Orthopedics, E-Da Dachang Hospital, Kaohsiung, Taiwan; 9 Department of Biomedical Engineering, I-Shou University, Kaohsiung, Taiwan; University of Ulsan College of Medicine, Asan Medical Center, REPUBLIC OF KOREA

## Abstract

**Background/objective:**

Osteoarthritis typically develops after surgery for traumatic fractures of the acetabulum and may result in total hip arthroplasty (THA). We conducted a population-based retrospective study to investigate the incidence of THA after treatment of acetabular, pelvic, and combined acetabular and pelvic fractures with open reduction-internal fixation surgery compared with that in the control group.

**Design:**

A retrospective population-based cohort study.

**Setting:**

Data were gathered from the Taiwan National Health Insurance Research Database.

**Participants:**

We enrolled 3041 patients with acetabular fractures, 5618 with pelvic fractures, and 733 with combined pelvic and acetabular fractures between January 1, 1997, and December 31, 2013, totaling 9392 individuals. The control group comprised 664,349 individuals. Study participants were followed up for the occurrence of THA until death or the end of the study period.

**Results:**

The THA rates after surgical intervention were 17.82%, 7.28%, and 18.01% in patients with acetabular, pelvic, and combined acetabular and pelvic fractures, respectively. Moreover, they were significantly higher for the acetabular fracture, pelvic fracture, and combined-fracture groups (adjusted hazard ratios [aHRs] = 58.42, 21.68, and 62.04, respectively) than for the control group (*p* < 0.0001) and significantly higher for the acetabular fracture and combined-fracture groups than for the pelvic fracture group (aHRs = 2.59 and 2.68, respectively; *p* < 0.0001).

**Conclusion:**

The incidence rates of THA after surgical intervention in the pelvic fracture, acetabular fracture, and combined-fracture groups were significantly higher than that of the control group.

## Introduction

Pelvic and acetabular fractures primarily result from high-energy trauma in relatively young patients (less than 40 year-old). In elderly patients, the cause may be low-energy trauma, such as that induced by minor falls.[1–3] Pelvic and acetabular fractures affect the pelvic ring structure and blood supply changes and may cause posttraumatic osteoarthritis and total hip arthroplasty (THA) for these patients.[4] Surgical intervention, particularly intra-articular procedures, is commonly recommended for acetabular fractures to facilitate recovery of joint congruency.[1],[5–8] Posttraumatic hip arthritis often occurs after acetabular fractures and is accelerated by malreduction during surgery. Furthermore, even when articular fractures can be treated with surgery, articular cartilage damage can subsequently cause osteoarthritic changes. The incidence of radiographic arthritis after acetabular fixation has been reported to be in the range of 20%–40%, with a subsequent THA rate of 8%–34%.[5,6]

Pelvic ring fractures are typically extra-articular hip fractures, but they may occur simultaneously with acetabular fractures involving the hip joint. The mechanisms of the relationship between this type of extra-articular fracture and traumatic osteoarthritis are still uncertain but may include vascularity changes after a fracture and structural changes engendering an asymmetric force distribution after the fracture[1,2,9,10] The outcomes of acetabular and pelvic fractures have primarily been reported in case series.[5–8,11,12] There are no data in the literature to support a direct increase in the risk to total hip replacement following pelvic ring fracture, but the complications from the surgical procedure may increase the risk of requiring THA. In one series, pelvic fracture resulted in long-term disability and painful leg length discrepancy affecting gait in a case of displaced pelvic fracture patterns.[10] No population studies have been conducted on the THA ratios required after pelvic and acetabular fracture fixation surgery.

Therefore, we conducted a population-based retrospective study to evaluate the incidence of THA following treatment of acetabular, pelvic, and combined acetabular and pelvic fractures with open reduction-internal fixation (ORIF) surgery compared with that in the control group.

## Materials and methods

### Patient and public involvement

#### No patients were involved in this study

The risks of requiring THA after pelvic and acetabular fractures have yet to be conclusively determined. The population-based cohort study was performed with the Taiwan National Health Insurance Research Database (NHIRD), which is provided by the National Health Insurance Administration (NHIA), Ministry of Health and Welfare and managed by the National Health Research Institutes. The interpretation and conclusions contained herein do not represent those of the NHIA, the Ministry of Health and Welfare or the National Health Research Institutes.

We enrolled patients who underwent surgery for pelvic, acetabular, or combined pelvic and acetabular fractures and subsequently received THA between January 1, 1997, and December 31, 2013. The associations of THA with various fractures (acetabular, pelvic, and combined) were investigated. The Taiwanese who were assessed were informed of their results after the study from the network of hospitals.

### Data sharing statement

Taiwan’s Ministry of Health and Welfare and the NHIA ensure the completeness and accuracy of the NHIRD. All data were fully anonymized before being extracted from the NHIA. All data are encrypted and may be analyzed only for academic research.

This retrospective population-based cohort study conducted longitudinal analysis on data recorded from the date of establishment of the NHIRD until the end of 2013. All individuals in the study sample were followed up for outcome identification according to the International Classification of Diseases, Ninth Revision, Clinical Modification (ICD-9-CM) codes. The data could be used within 5 years of receiving application approval from the NHIRD. All available data were used in this study, and no additional unpublished data were included. This study was approved by the Institutional Review Board of our hospital (EMRP-104-04) and the Taiwan NHIRD (NHIRD-104-167). This study was exempted from a full review by the Institutional Review Board of E-Da Hospital.

### Definition of study cohorts and outcomes

**Fig 1:**
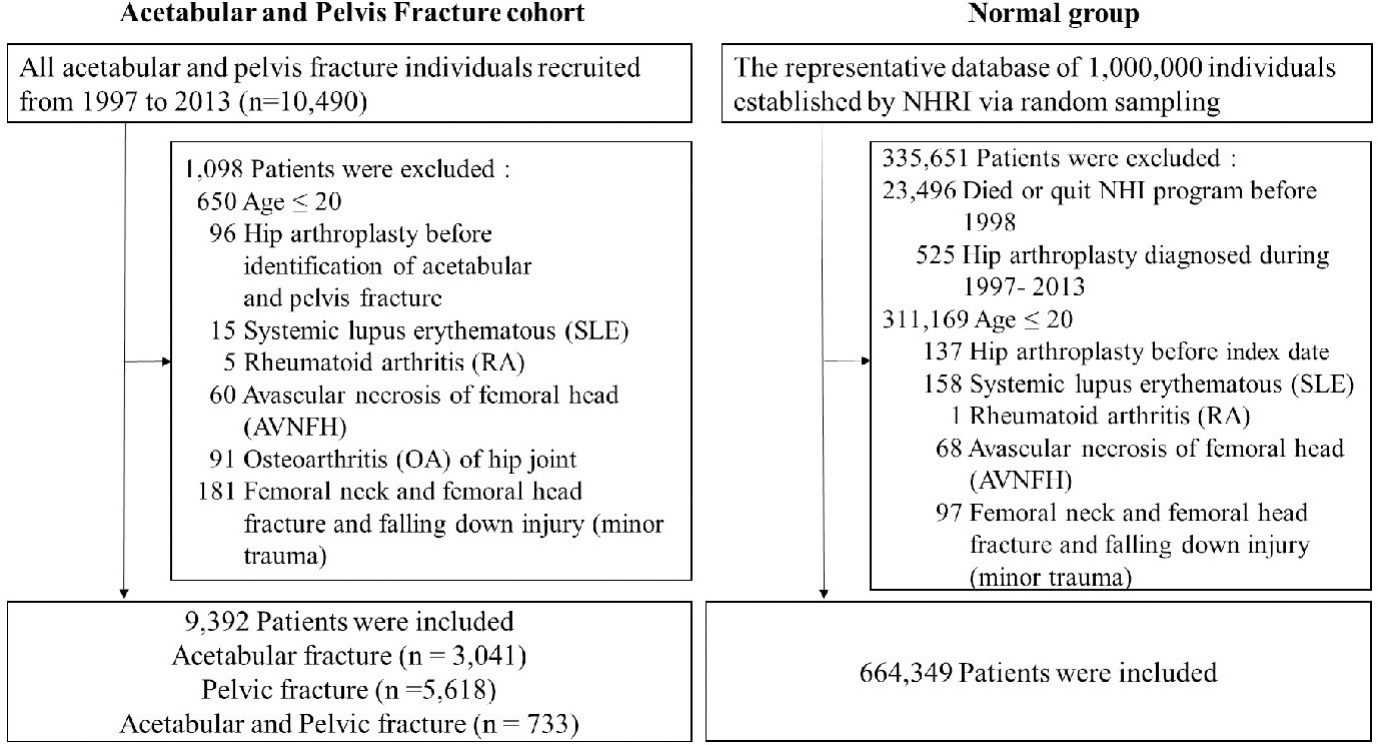
Study flow chart.

We included data from the NHIRD of patients who underwent surgical treatment for pelvic, acetabular, or combined pelvic and acetabular fractures between January 1, 1997, and December 31, 2013. We also included data from 1,000,000 people in the general population as a control group through random sampling from the NHIRD (Fig 1). The disease and surgical codes are listed in Appendix 1. The mean follow-up duration of the present study was 13.45 (range, 0.0192–16.91) years. We excluded the data of individuals who were younger than 20 years old, had systemic lupus erythematous, received THA before their fracture occurred, had rheumatoid arthritis, had femoral head avascular necrosis, had a history of femoral head or femoral neck fractures, or had developed osteoarthritis of the hip joint before the fracture occurred. The outcome of this study was defined as subsequent THA operations performed following index fracture surgery of the pelvis or acetabulum.

### Statistical analysis

Continuous variables were summarized as means and standard deviations, and categorical variables were listed as the number of cases and percentages. Continuous between-group variables were compared using the Student’s *t*-test, and categorical variables were assessed using either a chi-square test or Fisher’s exact test. Data were evaluated using a log-rank test and univariate and multivariate competing risk regression analyses. All statistical analyses were performed using SAS version 9.4 (SAS Institute, Cary, NC, USA). Two-sided *p* values of <0.05 were defined as statistically significant in this study.

## Results

### Baseline characteristics of patients

We included the data of 9392 patients. Group 1 comprised 3041 patients who had received ORIF surgery for acetabular fractures. This group included patients with acetabular fractures with or without pelvic fractures, but they received nonsurgical treatment for pelvic fractures. Group 2 comprised 5618 patients who had received ORIF surgery for pelvic fractures. This group included patients with pelvic fractures with or without acetabular fractures, but they received nonsurgical treatment for acetabular fractures. Group 3 comprised 733 patients who had received ORIF surgery for both pelvic and acetabular fractures simultaneously. These three groups of patients constituted the three study groups (Fig 1). There were 664,349 patients in the control group.

The baseline characteristics of the four groups are listed in Table 1. The average age of patients in the control group was 41.86 years, whereas those of patients in the acetabular fracture, pelvic fracture, and combined-fracture groups were 41.84, 42.79, and 42.76 years, respectively. Men constituted 80.59%, 63.34%, and 68.5% of the patients in the acetabular fracture, pelvic fracture, and combined-fracture groups, respectively.

**Table 1:** Comparison of demographic and clinical characteristics of the 4 groups.

		Normal (n = 664,349)		Acetabular fracture (n = 3,041)		Pelvic fracture (n = 5,618)		acetabular and pelvic fracture (n = 733)	P value
Age		41.86 ± 15.98		41.84 ± 14.90		42.79 ± 16.11		42.76 ± 15.07	<0.0001
Gender (Male) (%)		338921 (51.02)		2441 (80.59)		3532 (63.34)		498 (68.5)	<0.0001
CCI		0.09 ± 0.51		0.64 ± 1.45		0.76 ± 1.64		0.73 ± 1.45	<0.0001
Comorbidities									
Myocardial infarct		545 (0.08)		17 (0.56)		37 (0.66)		1 (0.14)	<0.0001
Congestive heart failure		1728 (0.26)		66 (2.17)		102 (1.82)		10 (1.36)	<0.0001
Peripheral vascular disease		493 (0.07)		33 (1.09)		87 (1.55)		10 (1.36)	<0.0001
Cerebrovascular disease		4478 (0.67)		90 (2.96)		246 (4.38)		26 (3.55)	<0.0001
Dementia		315 (0.05)		24 (0.79)		55 (0.98)		3 (0.41)	<0.0001
Chronic lung disease		3227 (0.49)		147 (4.83)		262 (4.66)		30 (4.09)	<0.0001
Connective tissue disease		461 (0.07)		26 (0.85)		87 (1.55)		12 (1.64)	<0.0001
Ulcer		6343 (0.95)		366 (12.04)		851 (15.15)		115 (15.69)	<0.0001
Chronic liver disease		3037 (0.46)		231 (7.6)		444 (7.9)		77 (10.5)	<0.0001
Diabetes		20302 (3.06)		288 (9.47)		499 (8.88)		79 (10.78)	<0.0001
Diabetes with end organ damage		1351 (0.2)		56 (1.84)		106 (1.89)		12 (1.64)	<0.0001
Hemiplegia		812 (0.12)		29 (0.95)		92 (1.64)		7 (0.95)	<0.0001
Moderate or severe kidney disease		3289 (0.5)		140 (4.6)		288 (5.13)		45 (6.14)	<0.0001
Malignant tumor		2650 (0.4)		45 (1.48)		133 (2.37)		10 (1.36)	<0.0001
Leukemia, lymphoma		155 (0.02)		3 (0.1)		4 (0.07)		1 (0.14)	0.0011
Moderate or severe liver disease		375 (0.06)		5 (0.16)		30 (0.53)		2 (0.27)	<0.0001
Metastasis		611 (0.09)		16 (0.53)		40 (0.71)		2 (0.27)	<0.0001
AIDS	-		-		-		-		-

### Comparison of three fracture groups and control group regarding incidence rates of subsequent THA

Table 2 lists the THA rates for the control group and three fracture groups. The THA rate in the control group was 0.43%; those in the acetabular, pelvic, and combined-fracture groups were 17.82%, 7.28%, and 18.01%, respectively.

**Table 2:** Comparison of numbers (%) and risk of hip arthroplasty between different 4 fracture group groups and normal group.

	Normal	Acetabular fracture	Pelvic fracture	acetabular and pelvic fracture	Total
Non-hip arthroplasty	661,488 (99.57)	2,499 (82.18)	5,209 (92.72)	601 (81.99)	669,797
Hip arthroplasty	2,861 (0.43)	542 (17.82)	409 (7.28)	132 (18.01)	3,944
Total	664,349	3,041	5,618	733	673,741

**Fig 2:**
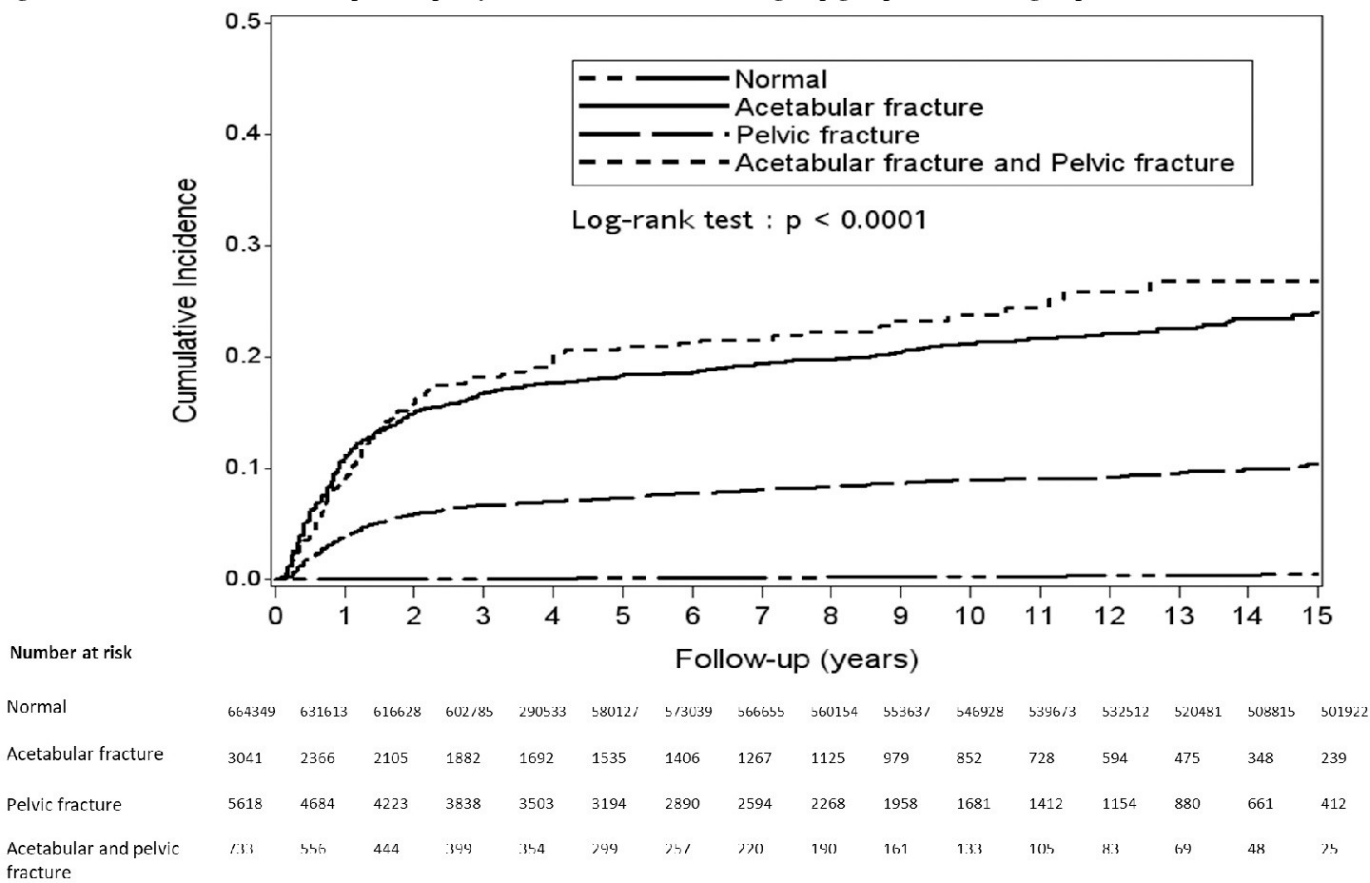
Cumulative incidence of hip arthroplasty of the three fracture groups and the control group.

The cumulative incidence of the control group showed a gradual, linear increase with time. The cumulative incidence of the three fracture groups increased curvilinearly with time for the first 2 years and gradually and linearly with time thereafter (Fig 2).

After 15 years, 0.5% (95% CI, 0%) of the control group cumulatively received THA. Compared with the control group, the acetabular fracture, pelvic fracture, and combined-fracture groups had a significantly higher incidence of THA (Fig 2), with 15-year cumulative incidence rates of 24% (95% CI, 22%–26%), 10.6% (95% CI, 9%–12%), and 26.8% (95% CI, 22%–32%), respectively (*p* < 0.0001). The acetabular fracture and combined-fracture groups also had significantly higher incidence rates of THA than the pelvic fracture group.

### Comparison of the three fracture groups and control group with respect to incidence of THA

The crude hazard ratios (HRs) and adjusted HRs (aHRs) of the four groups are presented in Table 3. The crude HR for the acetabular fracture group was 58.08%, whereas those of the pelvic fracture and combined-fracture groups were 22.42% and 64.9%, respectively (*p* < 0.0001). The aHRs for the acetabular fracture, pelvic fracture, and combined-fracture groups were 58.42%, 21.68%, and 62.04%, respectively (*p* < 0.0001).

**Table 3:** Risk of hip arthroplasty between different 4 fracture group groups and normal group.

	Crude HR (95% CI)	P value	Adjusted HR (95% CI)	P value
**Normal**	1.00	-		-
**Acetabular fracture**	58.08 (52.76–63.95)	< .0001	58.42 (52.65–64.81)	< .0001
**Pelvic fracture**	22.42 (20.18–24.92)	< .0001	21.68 (19.35–24.28)	< .0001
**Acetabular and pelvic fracture**	64.9 (54.01–78)	< .0001	62.04 (51.12–75.29)	< .0001
Age	1.03 (1.02–1.03)	< .0001	1.03 (1.03–1.03)	< .0001
Gender (Male vs. Female)	1.5 (1.41–1.6)	< .0001	1.21 (1.13–1.29)	< .0001
Comorbidities				
Myocardial infarct	2.85 (1.53–5.33)	0.001	0.7 (0.36–1.36)	0.2872
Congestive heart failure	4.47 (3.36–5.95)	< .0001	1.21 (0.88–1.68)	0.2471
Peripheral vascular disease	6.51 (4.27–9.93)	< .0001	1.09 (0.68–1.75)	0.7311
Cerebrovascular disease	2.81 (2.24–3.52)	< .0001	0.91 (0.7–1.18)	0.4629
Dementia	2.25 (0.94–5.41)	0.0699	0.28 (0.11–0.71)	0.0071
Chronic lung disease	4.08 (3.28–5.07)	< .0001	0.77 (0.6–0.99)	0.0449
Connective tissue disease	12.4 (8.97–17.14)	< .0001	2.36 (1.6–3.5)	< .0001
Ulcer	5.57 (4.86–6.37)	< .0001	1.07 (0.91–1.25)	0.4429
Chronic liver disease	6.59 (5.53–7.84)	< .0001	1.32 (1.07–1.63)	0.0085
Diabetes	2.06 (1.81–2.35)	< .0001	0.9 (0.78–1.04)	0.1555
Diabetes with end organ damage	3.63 (2.54–5.18)	< .0001	1.05 (0.69–1.61)	0.8158
Hemiplegia	2.79 (1.68–4.65)	< .0001	0.7 (0.41–1.21)	0.1987
Moderate or severe kidney disease	3.83 (3.06–4.78)	< .0001	0.86 (0.65–1.13)	0.2765
Malignant tumor	1.41 (0.93–2.12)	0.1042	0.54 (0.35–0.85)	0.0076
Leukemia, lymphoma	1.06 (0.15–7.54)	0.9541	0.53 (0.07–3.87)	0.5335
Moderate or severe liver disease	2.98 (1.41–6.29)	0.0042	1.12 (0.5–2.49)	0.7817
Metastasis	1.03 (0.38–2.74)	0.9589	0.46 (0.16–1.33)	0.1488

HR: Hazard Ratio. ratio; CI, confidence interval; NA, not applicable due to few events.

### Comparison of three fracture groups with respect to incidence of THA

Table 4 presents a comparison of THA incidence rates among the three fracture groups. The crude HRs for the pelvic fracture, acetabular fracture, and combined-fracture groups were 1, 2.56, and 2.72, respectively (*p* < 0.0001).

**Table 4:** Risk of hip arthroplasty between different 3 fracture group groups.

	Crude HR (95% CI)	P value	Adjusted HR (95% CI)	P value
**Pelvic fracture**	1.00	-		-
**Acetabular fracture**	2.56 (2.25–2.91)	< .0001	2.59 (2.27–2.96)	< .0001
**Acetabular and pelvic fracture**	2.72 (2.24–3.31)	< .0001	2.68 (2.2–3.27)	< .0001
Age	1.02 (1.01–1.02)	< .0001	1.02 (1.02–1.02)	< .0001
Gender (Male vs. Female)	1.36 (1.18–1.56)	< .0001	1.21 (1.05–1.4)	0.008
Comorbidities				
Myocardial infarct	1.11 (0.52–2.35)	0.7885	0.78 (0.35–1.7)	0.5231
Congestive heart failure	1.95 (1.39–2.73)	0.0001	1.47 (1.01–2.13)	0.042
Peripheral vascular disease	1.17 (0.71–1.94)	0.5307	0.96 (0.57–1.62)	0.8761
Cerebrovascular disease	1.29 (0.97–1.73)	0.0809	1 (0.72–1.37)	0.9783
Dementia	0.42 (0.15–1.12)	0.0814	0.3 (0.11–0.83)	0.0207
Chronic lung disease	1.33 (1.03–1.73)	0.0311	0.85 (0.63–1.13)	0.2577
Connective tissue disease	1.86 (1.2–2.88)	0.0054	1.63 (1.04–2.57)	0.0347
Ulcer	1.31 (1.12–1.54)	0.0008	1.09 (0.91–1.3)	0.3546
Chronic liver disease	1.38 (1.13–1.69)	0.0017	1.15 (0.92–1.44)	0.2065
Diabetes	1.48 (1.23–1.77)	< .0001	1.08 (0.87–1.34)	0.4642
Diabetes with end organ damage	1.47 (0.99–2.19)	0.0578	1.08 (0.67–1.76)	0.7424
Hemiplegia	0.81 (0.46–1.43)	0.4673	0.77 (0.43–1.38)	0.377
Moderate or severe kidney disease	1.2 (0.92–1.56)	0.1747	0.85 (0.62–1.16)	0.3018

### Risk of requiring total hip replacement at different time points

The annual incidence rates (per 1,000 patient-years) of total hip replacement within the first 2 years were 87.11 (70.64–106.27), 83.21 (75.29–-91.75), and 30.94 (27.49–34.70) among the combined, acetabular, and pelvic fracture groups (*p* < 0.0001 according to the exact Poisson test). Intriguingly, the annual incidence rates in the three groups were 21.37 (13.70–31.80), 14.04 (11.07–17.58), and 5.14 (3.90–6.66) between 2 and 5 years (*p* < 0.0001); 6.69 (2.69–13.78), 6.84 (4.91–9.28), and 3.69 (2.69–4.94) between 5 and 10 years (*p* < 0.0001); and 10.32 (2.81–26.43), 7.01 (4.22–10.94), and 2.50 (1.33–4.27) between 10 and 15 years (*p* < 0.0001). These findings further highlight that most total hip replacement surgeries were performed within 2 years of the index surgery.

## Discussion

Pelvic and acetabular fractures are relatively rare and typically result from high-energy traumas, such as injuries caused by motor vehicle accidents, falls from heights, and accidents during sports such as skiing.[1,2] On their first visit, such patients are nearly always sent to the emergency department and treated according to the advanced trauma life support principles. Subsequently, after patients’ vital signs and conditions stabilize, they receive definite treatment for the fractures.[13–18] Open reduction and internal fixation surgery is generally recommended for severe pelvic and acetabular fractures.[1,2,7,8] After open reduction and internal fixation surgery, mid-term and long-term results may be difficult to evaluate because of the means of transport adopted by patients in Taiwan to reach hospitals.[19–21] However, the long-term results of fracture surgery for hip joints are critical for patients in their life following the surgery.[22–25]

Because acetabular fractures embody an intra-articular fracture pattern, a combined treatment of open reduction and internal fixation is generally preferred. For pelvic fractures, the preferred treatments are diverse: For severe and unstable types of pelvic fractures, open reduction and internal fixation is the preferred treatment. However, for several partially unstable pelvic fractures, such as pubic ramus fractures, conservative treatment may be preferred.[26,27] We included only patients who received surgical treatment for pelvic and acetabular fractures. Malreduction with changing biomechanical force at the hip joints after fracture surgery and articular cartilage damage led to a relatively high likelihood of experiencing advanced traumatic osteoarthritis requiring subsequent THA.[15,22,28–32]

No previous study has focused on evaluating the risk of requiring THA after pelvic fractures. Studies on THA after acetabular fractures have reported incidence rates between 8% and 34%.[33,34] Our study population of 664,349 is among the largest cohorts used to evaluate subsequent THA after surgery for pelvic, acetabular, and combined pelvic and acetabular fractures.

In our cohort, men constituted 80.59%, 63.34%, and 68.5% of the acetabular, pelvic, and combined-fracture groups, respectively. A study demonstrated that pelvic and acetabular fractures occurred more frequently in men.[35] These rates may relate to the relatively reckless driving behavior of male drivers. The National Police Agency of Taiwan reported that 65% of traffic accidents in 2015 were caused by men (Table 1).

The incidence rate of THA for the pelvic fracture group was 7.28%. Results of the present study indicated that even individuals with pelvic fractures, which are an extra-articular fracture type, had a higher incidence of THA than individuals in the control group. Indeed, it appears that the hip joints were spared in the occurrences of extra-articular fractures, such as those occurring in the pubic rami, sacrum, or iliac alae. However, these injuries may still alter the physical mechanics of weight-bearing under pain, immobility, or positional changes. No data in the literature support a direct increase in the risk of total hip replacement, but the complications of the surgical procedure may increase the risk of requiring THA. In one series, pelvic fracture resulted in long-term disability and painful leg length discrepancy affecting gait in displaced pelvic fracture patterns.^13^ Several common complications were noted in the pelvic fracture group in this study, including ectopic ossification, postoperative infection, and nonunion and malunion of the pelvic ring, which can increase the risk of joint cartilage destruction. However, our study has some limitations and biases. Few patients from the pelvic fracture group had acetabular fracture for which no surgical treatment was received. The adverse effects of acetabular fractures in this group potentially increased the THA incidence rate.

In the acetabular fracture group, the incidence of THA was 17.82%. This result is similar to the incidence of THA after acetabular fractures reported in a Canadian study.[33,34] For the combined-fracture group, the incidence of THA was slightly higher, at 18.01%. The cumulative incidence of THA for all three fracture groups markedly increased (Fig 2) during the first 2 years, when the relationship between incidence and time was curvilinear. After 2 years, the incidence exhibited an almost linear increase with time.

The THA incidence rates of the three fracture groups were significantly higher than that of the control group (the HRs for the acetabular fracture, pelvic fracture, and combined-fracture groups were 58.42, 21.68, and 62.04, respectively [*p* < 0.0001]). The acetabular fracture group exhibited an incidence of THA more than twice that of the pelvic fracture group (the HRs for the acetabular fracture and combined-fracture groups were 2.56 and 2.76, respectively [*p* < 0.0001]). Acetabular fractures are intra-articular fractures that involve articular cartilage and result in relatively high risks of damage and the development of advanced osteoarthritis. Although acetabular fractures with gaps smaller than 2 mm have the potential to heal, the healed cartilage is fibrocartilage rather than hyaline cartilage and has less resistance to femoral head impaction[36].

### Limitations

Our data were acquired from Taiwan’s NHIRD, which registers only surgical fee codes. We did not evaluate results from computed tomography or plain radiography, which are processes that can lead to pelvic fracture, including pelvic ring injury (extra-articular fracture). Whether a relationship exists between this type of fracture and the occurrence of posttraumatic arthritis of the hip joint remains uncertain. Therefore, we could not analyze the patterns of the pelvic and acetabular fractures or the degrees to which various fracture patterns contributed to the incidence of THA. There were patients who were identified as having a pelvic fracture only; however, they might have had unknown accompanying acetabular fractures, which can produce biased results. However, the accuracy of diagnostic and surgical codes is strictly audited by the government because it is linked to payment and reimbursement.[37] This regulatory policy may help alleviate the concern of misclassification.

We acquired patient data for the period between January 1997 and December 2013. For studies on osteoarthritis, 15 years may be considered a long-term study period of the entire spectrum of the disease. We used the longest follow-up period of any related study. The results can accurately reflect the risks of developing posttraumatic hip osteoarthritis and requiring THA.

## Conclusion

The incidence rates of THA after surgical intervention for pelvic fractures, acetabular fractures, and combined-fractures were significantly greater (17.82%, 7.28%, 18.01%, respectively) than that of the control group (0.43%). Most THA procedures were performed less than 2 years after a fracture fixation. During the 2-year follow-up period, the incidence rates of THA in the pelvic fracture, acetabular fracture, and combined-fracture groups increased linearly with time.

## Supporting information

S1 Appendix ICD-9-CM codes and the corresponding diseases or procedures. (DOC)

## References

[1] Matta JM (1996) Fractures of the acetabulum: accuracy of reduction and clinical results in patients managed operatively within three weeks after the injury. J Bone Joint Surg Am 78: 1632–1645. 8934477

[2] Letournel E (2019) Acetabulum Fractures: Classification and Management. J Orthop Trauma 33 Suppl 2: S1–s2. 10.1097/BOT.0000000000001424 30688851

[3] Liebergall M, Mosheiff R, Low J, Goldvirt M, Matan Y, et al (1999) Acetabular fractures. Clinical outcome of surgical treatment. Clin Orthop Relat Res: 205–216. 10627737

[4] Feugier P, Fessy MH, Bejui J, Bouchet A (1997) Acetabular anatomy and the relationship with pelvic vascular structures. Implications in hip surgery. Surg Radiol Anat 19: 85–90. 10.1007/bf01628131 9210241

[5] Giannoudis PV, Grotz MR, Papakostidis C, Dinopoulos H (2005) Operative treatment of displaced fractures of the acetabulum. A meta-analysis. J Bone Joint Surg Br 87: 2–9. 15686228

[6] Dunet B, Tournier C, Billaud A, Lavoinne N, Fabre T, et al (2013) Acetabular fracture: long-term follow-up and factors associated with secondary implantation of total hip arthroplasty. Orthop Traumatol Surg Res 99: 281–290. 10.1016/j.otsr.2012.12.018 23562708

[7] Briffa N, Pearce R, Hill AM, Bircher M (2011) Outcomes of acetabular fracture fixation with ten years' follow-up. J Bone Joint Surg Br 93: 229–236. 10.1302/0301-620X.93B2.24056 21282764

[8] Brueton RN (1993) A review of 40 acetabular fractures: the importance of early surgery. Injury 24: 171–174. 10.1016/0020-1383(93)90285-e 8509186

[9] Manson TT, Reider L, O'Toole RV, Scharfstein DO, Tornetta P 3rd, et al (2016) Variation in Treatment of Displaced Geriatric Acetabular Fractures Among 15 Level-I Trauma Centers. J Orthop Trauma 30: 457–462. 10.1097/BOT.0000000000000632 27218694

[10] Tile M HD, Kellam JF (2003) Fractures of the Pelvis and Acetabulum; Philadelphia P, Lippincott Williams & Wilkins, editor.

[11] Katz JN, Losina E, Barrett J, Phillips CB, Mahomed NN, et al (2001) Association between hospital and surgeon procedure volume and outcomes of total hip replacement in the United States medicare population. J Bone Joint Surg Am 83-a: 1622–1629. 10.2106/00004623-200111000-00002 11701783

[12] Mears DC, Velyvis JH, Chang CP (2003) Displaced acetabular fractures managed operatively: indicators of outcome. Clin Orthop Relat Res: 173–186. 10.1097/00003086-200302000-00026 12567145

[13] Henry PD, Kreder HJ, Jenkinson RJ (2013) The osteoporotic acetabular fracture. Orthop Clin North Am 44: 201–215. 10.1016/j.ocl.2013.01.002 23544824

[14] Jain N, Pietrobon R, Hocker S, Guller U, Shankar A, et al (2004) The relationship between surgeon and hospital volume and outcomes for shoulder arthroplasty. J Bone Joint Surg Am 86-a: 496–505. 10.2106/00004623-200403000-00006 14996874

[15] O'Toole RV, Hui E, Chandra A, Nascone JW (2014) How often does open reduction and internal fixation of geriatric acetabular fractures lead to hip arthroplasty? J Orthop Trauma 28: 148–153. 10.1097/BOT.0b013e31829c739a 23719343

[16] Ravi B, Jenkinson R, Austin PC, Croxford R, Wasserstein D, et al (2014) Relation between surgeon volume and risk of complications after total hip arthroplasty: propensity score matched cohort study. Bmj 348: g3284 10.1136/bmj.g3284 24859902 PMC4032026

[17] Grimshaw CS, Moed BR (2010) Outcomes of posterior wall fractures of the acetabulum treated nonoperatively after diagnostic screening with dynamic stress examination under anesthesia. J Bone Joint Surg Am 92: 2792–2800. 10.2106/JBJS.J.00112 21123609

[18] Mears DC (1999) Surgical treatment of acetabular fractures in elderly patients with osteoporotic bone. J Am Acad Orthop Surg 7: 128–141. 10.5435/00124635-199903000-00006 10217820

[19] Bergin PF, Doppelt JD, Kephart CJ, Benke MT, Graeter JH, et al (2011) Comparison of minimally invasive direct anterior versus posterior total hip arthroplasty based on inflammation and muscle damage markers. J Bone Joint Surg Am 93: 1392–1398. 10.2106/JBJS.J.00557 21915544 PMC3143583

[20] Gary JL, Lefaivre KA, Gerold F, Hay MT, Reinert CM, et al (2011) Survivorship of the native hip joint after percutaneous repair of acetabular fractures in the elderly. Injury 42: 1144–1151. 10.1016/j.injury.2010.08.035 20850738

[21] McIntosh AL, Hanssen AD, Wenger DE, Osmon DR (2006) Recent intraarticular steroid injection may increase infection rates in primary THA. Clin Orthop Relat Res 451: 50–54. 10.1097/01.blo.0000229318.51254.79 16906098

[22] Hayes PJ, Carroll CM, Roberts CS, Seligson D, Lau E, et al (2013) Operative treatment of acetabular fractures in the Medicare population. Orthopedics 36: e1065–1070. 10.3928/01477447-20130724-25 23937755

[23] Kreder HJ, Rozen N, Borkhoff CM, Laflamme YG, McKee MD, et al (2006) Determinants of functional outcome after simple and complex acetabular fractures involving the posterior wall. J Bone Joint Surg Br 88: 776–782. 10.1302/0301-620X.88B6.17342 16720773

[24] Murphy D, Kaliszer M, Rice J, McElwain JP (2003) Outcome after acetabular fracture. Prognostic factors and their inter-relationships. Injury 34: 512–517. 10.1016/s0020-1383(02)00349-2 12832177

[25] Wright R, Barrett K, Christie MJ, Johnson KD (1994) Acetabular fractures: long-term follow-up of open reduction and internal fixation. J Orthop Trauma 8: 397–403. 10.1097/00005131-199410000-00005 7996322

[26] Tile M (1988) Pelvic ring fractures: should they be fixed? J Bone Joint Surg Br 70: 1–12. 3276697 10.1302/0301-620X.70B1.3276697

[27] Tornetta P 3rd (2001) Displaced acetabular fractures: indications for operative and nonoperative management. J Am Acad Orthop Surg 9: 18–28. 10.5435/00124635-200101000-00003 11174160

[28] Anglen JO, Burd TA, Hendricks KJ, Harrison P (2003) The "Gull Sign": a harbinger of failure for internal fixation of geriatric acetabular fractures. J Orthop Trauma 17: 625–634. 10.1097/00005131-200310000-00005 14574190

[29] Bastian JD, Tannast M, Siebenrock KA, Keel MJ (2013) Mid-term results in relation to age and analysis of predictive factors after fixation of acetabular fractures using the modified Stoppa approach. Injury 44: 1793–1798. 10.1016/j.injury.2013.08.009 24008225

[30] Ferguson TA, Patel R, Bhandari M, Matta JM (2010) Fractures of the acetabulum in patients aged 60 years and older: an epidemiological and radiological study. J Bone Joint Surg Br 92: 250–257. 10.1302/0301-620X.92B2.22488 20130318

[31] Laird A, Keating JF (2005) Acetabular fractures: a 16-year prospective epidemiological study. J Bone Joint Surg Br 87: 969–973. 10.1302/0301-620X.87B7.16017 15972913

[32] Tannast M, Najibi S, Matta JM (2012) Two to twenty-year survivorship of the hip in 810 patients with operatively treated acetabular fractures. J Bone Joint Surg Am 94: 1559–1567. 10.2106/JBJS.K.00444 22992846

[33] Henry PDG, Si-Hyeong Park S, Paterson JM, Kreder HJ, Jenkinson R, et al (2018) Risk of Hip Arthroplasty After Open Reduction Internal Fixation of a Fracture of the Acetabulum: A Matched Cohort Study. J Orthop Trauma 32: 134–140. 10.1097/BOT.0000000000001048 29462122

[34] Juurlink D, Preyra C, Croxford R, Chong A, Austin P, et al (2006) Canadian institute for health information discharge abstract database: a validation study. ICES investigative report Institute for Clinical Evaluative Sciences, Toronto.

[35] Demetriades D, Karaiskakis M, Toutouzas K, Alo K, Velmahos G, et al (2002) Pelvic fractures: epidemiology and predictors of associated abdominal injuries and outcomes. J Am Coll Surg 195: 1–10. 10.1016/s1072-7515(02)01197-3 12113532

[36] Frank RM, Cotter EJ, Hannon CP, Harrast JJ, Cole BJ (2019) Cartilage Restoration Surgery: Incidence Rates, Complications, and Trends as Reported by the American Board of Orthopaedic Surgery Part II Candidates. Arthroscopy 35: 171–178. 10.1016/j.arthro.2018.08.028 30611347

[37] Hsu Y-C (2015) Analyzing Taiwan's National Health Insurance Research Database to explicate the allocation of health-care resources. source: Advances in Digestive Medicine 2.

