# Supplementary material for: Long-term total hip arthroplasty rates in patients with acetabular and pelvic fractures after surgery: A population-based cohort study
Source: PLoS One. 2020 Apr 3;15(4):e0231092. doi: 10.1371/journal.pone.0231092 (PMC7122785; doi:10.1371/journal.pone.0231092)
Supplement: S1 Appendix — (DOC) [file pone.0231092.s001.doc]

**Appendix 1. ICD-9-CM codes and the corresponding diseases or procedures**

| **Disease or procedures** | **Corresponding ICD-9-CM codes** |
| --- | --- |
| Acetabular fracture | 808.0, 808.1 |
| Pelvic fracture | 808.2, 808.3, 808.4x, 808.5y, 808.8, 808.9 |
|  |  |
| Open reduction internal fixation surgeries for |  |
| Acetabulum | 64236B |
| Pelvis | 64161B |
|  |  |

Footnotes: ICD-9-CM, International Classification of Diseases, 9th Revision, Clinical Modification;
